# Several Alphaherpesviruses Interact Similarly with the NF-κB Pathway and Suppress NF-κB-Dependent Gene Expression

**DOI:** 10.1128/spectrum.01421-23

**Published:** 2023-07-19

**Authors:** Nicolás Romero, Alexander Tishchenko, Ruth Verhamme, Shelly M. Wuerzberger-Davis, Cliff Van Waesberghe, Hans J. Nauwynck, Shigeki Miyamoto, Herman W. Favoreel

**Affiliations:** a Department of Translational Physiology, Infectiology and Public Health–Faculty of Veterinary Medicine, Ghent University, Ghent, Belgium; b Department of Microbiology, Blavatnik Institute, Harvard Medical School, Boston, Massachusetts, USA; c McArdle Laboratory for Cancer Research, Department of Oncology, University of Wisconsin-Madison, Madison, Wisconsin, USA; Oklahoma State University College of Veterinary Medicine

**Keywords:** NF-κB, alphaherpesvirus, bovine alphaherpesvirus 1, epithelial cells, feline alphaherpesvirus 1, herpes simplex virus, pseudorabies virus

## Abstract

Alphaherpesvirus infection is associated with attenuation of different aspects of the host innate immune response that is elicited to confine primary infections at the mucosal epithelia. Here, we report that infection of epithelial cells with several alphaherpesviruses of different species, including herpes simplex virus 1 and 2 (HSV-1 and HSV-2), feline alphaherpesvirus 1 (FHV-1), and bovine alphaherpesvirus 1 (BoHV-1) results in the inactivation of the responses driven by the nuclear factor kappa B (NF-κB) pathway, considered a pillar of the innate immune response. The mode to interact with and circumvent NF-κB-driven responses in infected epithelial cells is seemingly conserved in human, feline, and porcine alphaherpesviruses, consisting of a persistent activation of the NF-κB cascade but a potent repression of NF-κB-dependent transcription activity, which relies on replication of viral genomes. However, BoHV-1 apparently deviates from the other investigated members of the taxon in this respect, as BoHV-1-infected epithelial cells do not display the persistent NF-κB activation observed for the other alphaherpesviruses. In conclusion, this study suggests that inhibition of NF-κB transcription activity is a strategy used by several alphaherpesviruses to prevent NF-κB-driven responses in infected epithelial cells.

**IMPORTANCE** The current study provides a side-by-side comparison of the interaction of different alphaherpesviruses with NF-κB, a key and central player in the (proinflammatory) innate host response, in infected nontransformed epithelial cell lines. We report that all studied viruses prevent expression of the hallmark NF-κB-dependent gene IκB, often but not always via similar strategies, pointing to suppression of NF-κB-dependent host gene expression in infected epithelial cells as a common and therefore likely important aspect of alphaherpesviruses.

## INTRODUCTION

*Alphaherpesvirinae* is the largest subfamily of the herpesviruses and comprises viral pathogens that infect a broad diversity of mammals, but also birds and reptiles. Herpes simplex virus 1 and 2 (HSV-1 and HSV-2) and varicella zoster virus (VZV) in humans, porcine pseudorabies virus (PRV), bovine alphaherpesvirus 1 (BoHV-1), and feline alphaherpesvirus 1 (FHV-1) all belong to this subfamily. The evolution of alphaherpesviruses has occurred in tight connection with animal speciation, given that, contrary to what has been observed for other virus taxa, cross-species transmission events were relatively infrequent (1–3). As a consequence, alphaherpesviruses have acquired a remarkable set of tools to suppress and/or evade the antiviral responses of their host, which has resulted in a fine-tuned viral dominance over their natural hosts. Of note, suppression of antiviral innate responses at the initial sites of alphaherpesvirus replication, the mucosal epithelia, is critically important for successful colonization of host individuals (4, 5).

The proinflammatory NF-κB signaling pathway is at the heart of the innate defense against pathogens, including viruses, and is activated, e.g., upon recognition of pathogen-associated molecular patterns via pattern recognition receptors. Briefly, activation of the canonical NF-κB relies on the proteasomal degradation of the NF-κB inhibitory protein IκBα, allowing subsequent nuclear import of NF-κB subunits to the cell nucleus to trigger transcription of a particular subset of genes (6–8). We and others have reported that, although infection of epithelial cells with alphaherpesviruses typically results in activation of NF-κB signaling, different alphaherpesviruses suppress the default NF-κB activation pathway and the (proinflammatory) consequences of this pathway (9–11).

The interaction of alphaherpesviruses with NF-κB signaling in infected epithelial cells is mainly documented for HSV-1 and, to a lesser extent, PRV (9, 12, 13). HSV-1 infection triggers activation of the NF-κB cascade in epithelial cells, apparently in a biphasic manner and involving several viral proteins (10, 11, 14–19). After a first, temporal wave of NF-κB activation that is elicited by, e.g., envelope and tegument proteins of virus particles during virus attachment to and entry in host cells, the NF-κB pathway is again activated later in infection in an aberrant and persistent manner consisting of the continued degradation of the NF-κB inhibitory protein IκBα and binding of NF-κB to DNA (10, 11, 20, 21). Despite this biphasic activation, a number of HSV-1 proteins have been identified as inhibitors of NF-κB activation (22–27), indicating that HSV-1-induced NF-κB activation is likely the product of a complex balance between activating and repressing mechanisms. Despite activation of the pathway, HSV-1 infection disables NF-κB-dependent host gene transcription (10, 11, 21), although some reports claim that HSV-1 infection does lead to a productive NF-κB activation that has a proviral effect by preventing infected cells from undergoing apoptotic cell death and favoring virus spread (28, 29). Interestingly, activation of the NF-κB pathway by the closely related HSV-2 does appear to lead to NF-κB-driven host responses (30, 31).

More recently, we found that PRV infection of immortalized as well as primary porcine epithelial cells leads to a gradual and unceasing degradation of the inhibitory IκBα protein and concomitant persistent nuclear translocation of NF-κB. However, despite this continuous activation of the pathway, NF-κB-dependent gene expression is blocked at the transcriptional level in the cell nucleus (9, 12, 13).

Whereas activation of the NF-κB pathway appears to be a general host response upon epithelial cell infection by alphaherpesviruses, inhibition of the NF-κB pathway and/or its consequences appears to also be a quite general feature of these pathogens and is therefore likely important in the context of *in vivo* infections at epithelial surfaces. At the same time, apparent differences in the strategies used by alphaherpesviruses to block productive NF-κB pathway activation suggest that at least some of these mechanisms were independently acquired in evolution, as a paradigm of convergent evolution. However, no study so far has directly compared the interaction of alphaherpesviruses of different species with the NF-κB signaling pathway in epithelial cells, complicating comparison and interpretation of studies of different alphaherpesviruses. In addition, very little is known about the interaction of nonhuman or nonporcine alphaherpesviruses with the NF-κB pathway.

Therefore, in this paper, we aimed to explore to what extent different alphaherpesviruses (i.e., HSV-1, HSV-2, BoHV-1, and FHV-1) interact similarly and/or differently with this chief and very conserved signaling pathway of the innate immune system (6, 7). In all cases, we use infected epithelial cells that are used commonly in studies of the respective pathogens, to minimize the variability corresponding to cell lineage and to increase the applicability of the results to commonly used virus-host cell interaction models. In addition, we avoided the use of tumor cell lines, given that NF-κB signaling is often dysregulated in cancer cells independently of the virus infection (32). Four parameters were used to define the status of the NF-κB pathway: (i) the degradation of the inhibitory IκBα protein and (ii) nuclear import of the NF-κB p65 subunit were used as indicators of NF-κB cascade activation, and in case of turning positive if further analyzed, (iii) the ability of NF-κB complexes to interact *in vitro* with κB binding sites and (iv) transcription of the IκBα gene as a hallmark of NF-κB-dependent gene expression were used (7).

Our findings show remarkable similarities and divergences in the way these viruses activate and restrain the NF-κB-pathway in epithelial cells. In all cases, alphaherpesvirus infection did not appear to result in productive activation of the NF-κB pathway, emphasizing the likely importance of suppression of the consequences of NF-κB signaling in epithelia infected by alphaherpesviruses. Furthermore, notable mechanistic differences were seen in the interaction with NF-κB for viral species that share high conservation in terms of genome sequence (e.g., BoHV-1 versus PRV, HSV-1 versus HSV-2), suggesting a very dynamic evolution in the mechanisms used by alphaherpesviruses to interfere with the activity of the NF-κB pathway.

## RESULTS

### BoHV-1 infection of bovine kidney epithelial cells does not lead to activation of the NF-κB signaling cascade.

**FIG 1:**
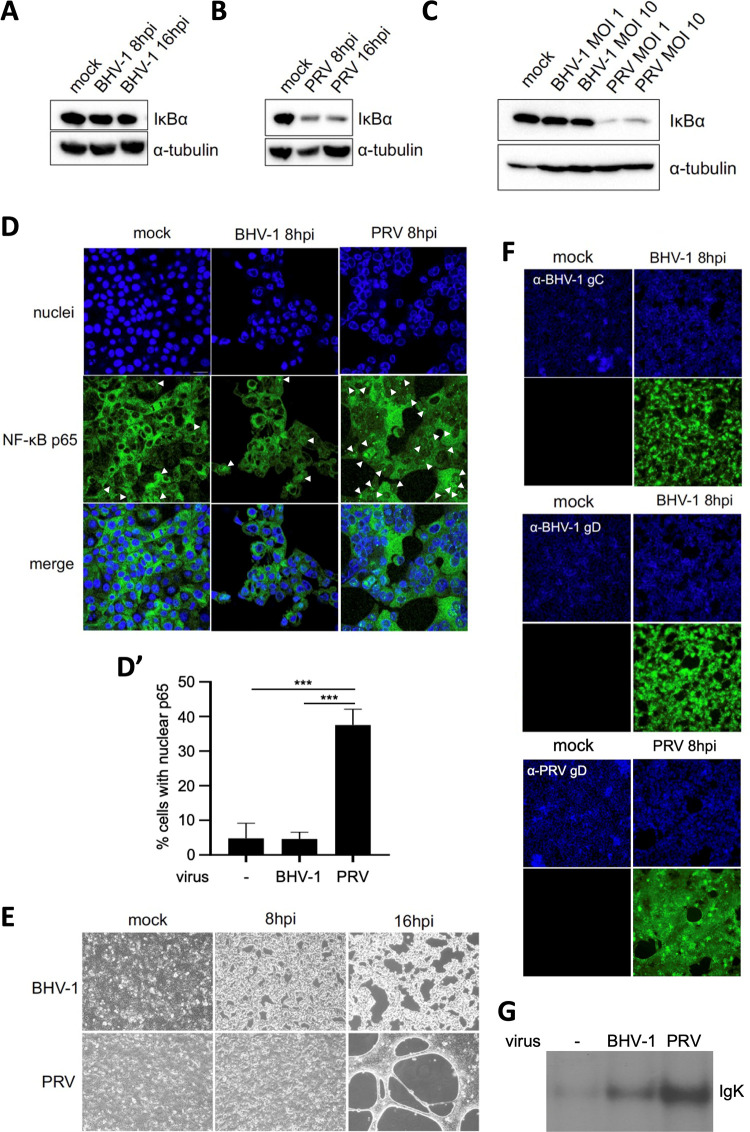
Interaction of BoHV-1 with the NF-κB pathway in bovine kidney epithelial cells. (A to C) Western blot analysis of the IκBα protein in BoHV-1-infected (A) and PRV-infected (B) MDBK cells at 8 and 16 hpi (MOI of 10 PFU/cell) or in BoHV-1- or PRV-infected MDBK cells at 8 hpi using an MOI of either 1 PFU/cell or 10 PFU/cell (C). (D and D′) Immunofluorescence detection of the NF-κB p65 subunit in BoHV-1- and PRV-infected MDBK cells at 8 hpi (MOI of 10 PFU/cell). NF-κB p65 is represented in green, and cell nuclei are shown in blue. Arrowheads indicate cells with nuclear p65. The graph in panel D′ shows the mean and standard deviation of three independent replicates. Asterisks indicate statistically significant differences (***, *P* < 0.001). (E) Light microscopy images showing the cytopathic effect caused by BoHV-1 and PRV infection in MDBK cell monolayers at 8 and 16 hpi (MOI of 10 PFU/cell). (F) Immunofluorescence of viral glycoproteins in MDBK cells infected with BoHV-1 (gC and gD) and PRV (gD) at 8 hpi (MOI of 10 PFU/cell). Viral glycoproteins are shown in green, and the cell nuclei is shown in blue. All assays were independently repeated three times. (G) NF-κB EMSA assessing the *in vitro* interaction of NF-κB transcription factors with κB sequences using mock-infected MDBK cells, BoHV-1-infected MDBK cells at 16 hpi (MOI of 10 PFU/cell), or PRV-infected MDBK cells at 16 hpi (MOI of 10 PFU/cell). BoHV-1 is indicated as BHV-1 in the figure to save space.

A hallmark of activation of the NF-κB signaling pathway is the degradation of the IκBα protein, the inhibitor protein associated with cytoplasmic NF-κB that needs to be degraded to allow NF-κB to migrate to the nucleus. To investigate whether BoHV-1 infection of Madin Darby bovine kidney (MDBK) epithelial cells trigger activation of the NF-κB signaling pathway, we assessed the degradation of the IκBα protein. As shown in Fig. 1A, Western blotting shows that BoHV-1 infection does not induce IκBα degradation at either 8 h postinfection (hpi) or 16 hpi using a high multiplicity of infection (MOI; 10 PFU/cell). This lack of BoHV-1-induced NF-κB activation in MDBK cells is not due to cell type-specific peculiarities with regard to IκBα degradation and/or NF-κB activation, as we showed earlier (9) and confirmed here (Fig. 1B) that infection of MDBK cells with the porcine alphaherpesvirus PRV does result in degradation of the IκBα protein. Comparable results were obtained when using either an MOI of 1 or an MOI of 10 PFU/cell in BoHV-1- and PRV-infected MDBK cells at 8 hpi (Fig. 1C). In line with these results, we found that the p65 subunit of NF-κB, which migrates to the nucleus upon NF-κB activation, remains largely cytoplasmic in BoHV-1-infected MDBK cells, with a largely comparable subcellular p65 distribution in BoHV1- and mock-infected cells (Fig. 1D). Quite to the contrary, PRV-infected MDBK cells at 8 hpi do show substantial and significantly increased nuclear import of NF-κB p65, in line with our earlier data (Fig. 1D) (9, 12, 13). In addition, the selected time points of analysis (8 hpi and 16 hpi) correspond to a very prominent virus-induced cytopathic effect, in both BoHV-1- and PRV-infected MDBK cells (Fig. 1E), indicating that BoHV-1 efficiently infected the MDBK cells. In line with this, a strong accumulation of viral late proteins (BoHV-1 gC and gD and PRV gD) was detected by immunofluorescence at 8 hpi in virtually all MDBK cells, confirming uniform infection and expression of late viral proteins by that time point (Fig. 1F). These data suggest that BoHV-1 infection does not trigger substantial activation of the NF-κB pathway. As an additional assay to assess this, we performed electrophoretic mobility shift assays (EMSAs), which allow the detection of activated NF-κB that can bind to so-called κB-binding sequences, the NF-κB target sequences in DNA. EMSAs (Fig. 1G) indicate noticeable but weak binding of NF-κB from BoHV-1-infected MDBK to κB-binding sequences, whereas NF-κB from PRV-infected MDBK cells binds prominently to κB-binding sequences. Together, these data show that BoHV-1 infection does not lead to prominent activation of the NF-κB pathway in MDBK cells, possibly by either not triggering it effectively or by blocking the signaling cascade at the level of IκBα degradation and/or at upstream step(s). As such, although PRV and BoHV-1 are evolutionarily closely related (3), both viruses show notable differences in their interaction with the NF-κB pathway in infected epithelial cells.

### FHV-1 infection of feline kidney epithelial cells activates the NF-κB signaling axis but suppresses NF-κB-dependent gene expression.

**FIG 2:**
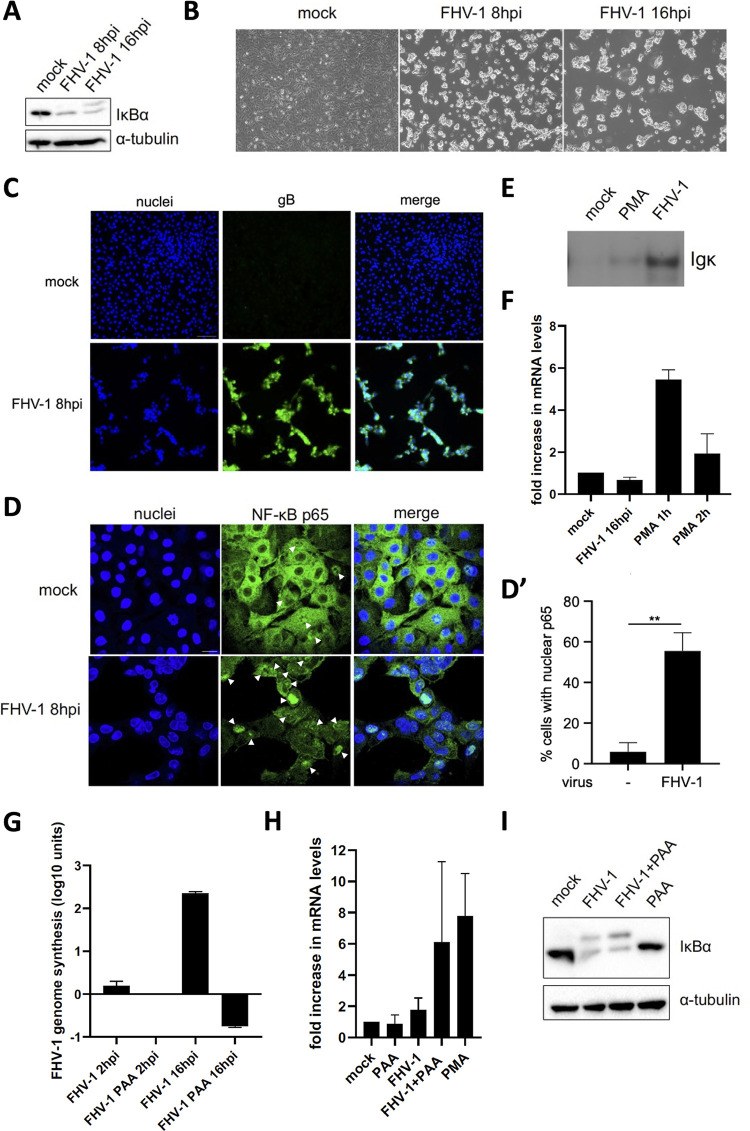
Interaction of FHV-1 with the NF-κB pathway in feline kidney epithelial cells. (A) Western blot analysis of IκBα protein degradation in FHV-1-infected CRFK cells at 8 and 16 hpi (MOI of 10 PFU/cell). (B) Light microscopy images indicating the cytopathic effect caused by FHV-1 in CRFK cell monolayers at 8 and 16 hpi (MOI of 10 PFU/cell). (C) Immunofluorescence images of mock-infected and FHV-1-infected CRFK cells at 8 hpi (MOI of 10 PFU/cell). Viral gB protein is shown in green, and cell nuclei are shown in blue. (D and D′) Immunofluorescence detection of the NF-κB p65 subunit in FHV-1-infected CRFK cells at 8 hpi (MOI of 10 PFU/cell). NF-κB p65 is shown in green, and cell nuclei are shown in blue. Arrowheads indicate cells with nuclear p65. The graphs in panel D′ shows the mean and standard deviation of three independent replicates. Asterisks indicate statistically significant differences (**, *P* < 0.01). (E) NF-κB EMSA assessing the *in vitro* interaction of NF-κB transcription factors with κB sequences using mock-infected CRFK cells, FHV-1-infected CRFK cells at 16 hpi (MOI of 10 PFU/cell), or PMA-treated CRFK cells (1 h, 20 ng/mL). (F) RT-qPCR-based IκBα mRNA levels in FHV-1-infected CRFK cells at 16 hpi (MOI of 10 PFU/cell) or treated with PMA for 1 h or 2 h (20 ng/mL). The graph indicates the mean and standard deviation of the relative fold change in IκBα mRNA abundance in comparison with mock-infected CRFK cells based on three independent repeats of the experiment (transcript levels were normalized to 18S rRNA levels). (G) qPCR-based intracellular viral DNA loads in FHV-1-infected CRFK cells at 2 and 16 hpi (MOI of 10 PFU/cell) in the absence or presence of 400 μg/mL PAA. The graph illustrates the mean and standard deviation (in log_10_ units) of the fold difference in FHV-1 genome levels compared to PAA-treated FHV-1-infected CRFK cells at 2 hpi based on three independent repeats of the assay (data were normalized to cellular genome copies based on the feline β-2-microglobulin [B2M] gene). Values were relative to PAA-treated PRV-infected cells at 2 hpi. (H) RT-qPCR-based assessment of IκBα transcripts in CRFK cells treated or not with PAA (400 μg/mL), either infected or not with FHV-1, and harvested at 16 hpi (MOI of 10 PFU/cell). Treatment of CRFK cells with PMA for 1 h (20 ng/mL) was used as a positive control to trigger IκBα transcription. The graph indicates the mean and standard deviation of the fold change in IκBα mRNA loads with respect to mock-infected CRFK cells based on three independent repeats of the experiment (transcript levels were normalized to 18S rRNA levels). (I) Western blot analysis of the IκBα in mock- and FHV-1-infected CRFK cells in the presence or absence of PAA (400 μg/mL) at 16 hpi (MOI of 10 PFU/cell). PAA was added to CRFK cells 30 min before virus inoculation and kept throughout infection. All assays were independently repeated three times.

Similar to the assays on BoHV-1, we assessed whether FHV-1 infection of epithelial Crandell-Rees feline kidney (CRFK) cells triggers degradation of the IκBα protein at 8 hpi and/or 16 hpi. Fig. 2A shows that IκBα is degraded in FHV-1-infected CRFK cells at both time points that coincide with prominent cytopathic effect (CPE) (Fig. 2B). In addition, immunofluorescence assays confirmed that virtually all cells were infected and expressed the viral glycoprotein gB at 8 hpi (Fig. 2C). In line with this, FHV-1-induced IκBα degradation correlates with significantly increased nuclear translocation of NF-κB p65 in infected CRFK cells at 8 hpi (Fig. 2D). Since these data indicate that FHV-1 infection triggers activation of the NF-κB pathway, an important question is whether this leads to the generation of activated NF-κB that can bind to so-called κB-binding sequences, the NF-κB target sequences in DNA. Hence, EMSAs were performed to determine whether activated NF-κB in cell lysates of FHV-1-infected cells binds to κB-binding sequences. Fig. 2E shows that indeed, at least *in vitro*, activated NF-κB in FHV-1-infected CRFK cells binds to κB-binding sequences. Finally, we assessed by real-time quantitative (RT-qPCR) whether NF-κB activation in FHV-1-infected CRFK cells leads to expression of the hallmark NF-κB-driven gene, the IκBα gene (33, 34). Interestingly, FHV-1 infection of CRFK did not lead to increased expression of IκBα mRNA (Fig. 2F), indicating that the virus suppresses the transcriptional consequences of NF-κB activation. Treatment of CRFK cells with the general NF-κB inducer phorbol 12-myristate 13-acetate (PMA) served as the positive control for the induction of IκBα gene transcription, which triggered prominent IκBα expression (Fig. 2F) despite triggering weaker NF-κB activation compared to FHV-1 infection based on the EMSA results (Fig. 2E). Overall, these data on FHV-1-infected epithelial cells are very much in line with our earlier results in PRV-infected epithelial cells (9, 12, 13). In PRV, we found that virus-induced repression of NF-κB-driven gene expression can be relieved by inhibition of viral DNA replication using the viral DNA polymerase inhibitor phosphonoacetic acid (PAA) (12). To assess whether this is also the case in FHV-1, we first confirmed by quantitative PCR (qPCR) that PAA effectively suppresses viral DNA replication in FHV-1-infected CRFK cells (Fig. 2G). Subsequent RT-qPCR assays showed that treatment of FHV-1-infected CRFK cells with PAA led to an increase in IκBα gene transcription (Fig. 2H). Although the increase did not reach statistical significance, it was reproducible and observed in each of the three independent repeats. This suggests that, comparable to PRV infection, suppression of NF-κB-dependent IκBα gene transcription by FHV-1 (at least in part) depends on replication of the viral genome. Importantly, however, the PAA-dependent increase in NF-κB-dependent IκBα gene transcription in FHV-1-infected CRFK cells does not correlate with a replenishment of IκBα protein levels at 16 hpi (Fig. 2I), unlike what is observed in PRV-infected epithelial cells (12), indicating that in FHV-1-infected cells, additional mechanisms may prevent IκBα mRNA translation to protein and/or lead to persistent degradation of the IκBα protein.

In conclusion, FHV-1 infection of CRFK cells triggers activation of the NF-κB signaling cascade but blocks the NF-κB-driven responses at the transcriptional level, which largely corresponds to how PRV modulates this key innate signaling pathway in infected epithelial cells.

### HSV-1 and HSV-2 infection of primate kidney epithelial cells activates the NF-κB signaling axis, but both viruses display differences in their interaction with NF-κB.

**FIG 3:**
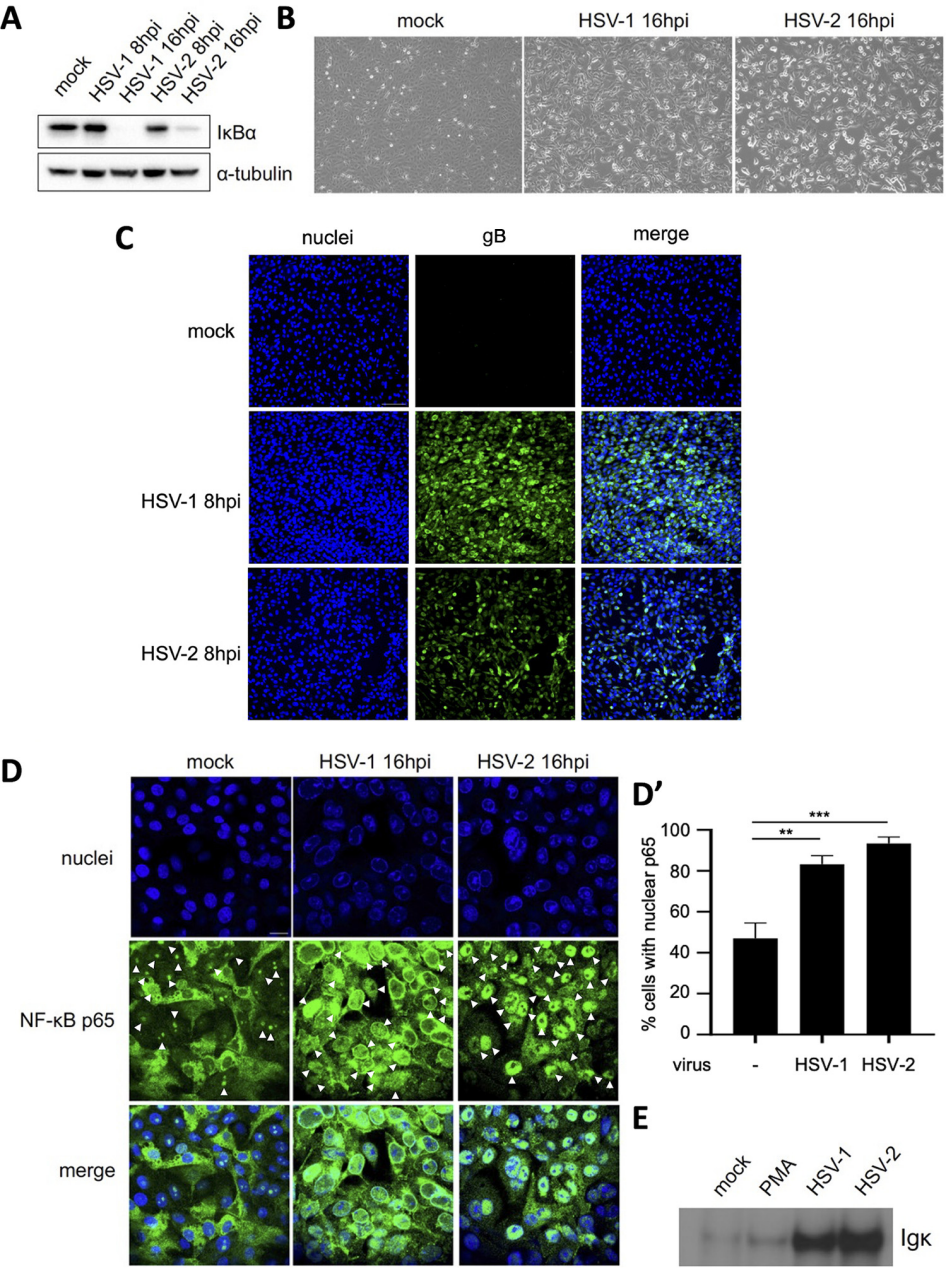
HSV-1 and HSV-2 trigger NF-κB activation in Vero epithelial cells. (A) Western blot analysis of IκBα protein degradation in Vero cells infected with HSV-1 and HSV-2 at 8 and 16 hpi (MOI of 10 PFU/cell). (B) Light microscopy images of HSV-1- and HSV-2-infected Vero cells at 16 hpi (MOI of 10 PFU/cell). (C) Immunofluorescence images of mock-infected and HSV-1- or HSV-2-infected Vero cells at 8 hpi (MOI of 10 PFU/cell). Viral gB protein is shown in green, and cell nuclei are shown in blue. (D and D′) Immunofluorescence analyses of NF-κB p65 localization in Vero cells infected with HSV-1 and HSV-2 at 16 hpi (MOI of 10 PFU/cell). Arrowheads indicate cells with nuclear p65. The graph in D′ shows the mean and standard deviation of three independent replicates. Asterisks indicate statistically significant differences (**, *P* < 0.01; ***, *P* < 0.001). (E) NF-κB EMSA assessing the *in vitro* interaction of NF-κB transcription factors with κB sequences using mock-infected Vero cells, HSV-1- or HSV-2-infected Vero cells at 16 hpi (MOI of 10 PFU/cell), or PMA-treated Vero cells (1 h, 20 ng/mL).

Vero primate kidney epithelial cells are the most widely used cell line in HSV research, which is why we opted to assess the impact of HSV-1 and HSV-2 infection on NF-κB signaling in this cell type. Again, we first explored the capacity of HSV-1 and -2 to trigger IκBα protein degradation in infected Vero cells at 8 and 16 hpi. As shown in Fig. 3A, infection with both types of HSV leads to IκBα degradation by 16 hpi, a time point that corresponds with noticeable HSV-induced CPE (Fig. 3B). In addition, immunofluorescence assays confirmed that virtually all cells were infected and expressed the viral glycoprotein gB at 8 hpi (Fig. 3C). When assessing nuclear translocation of the p65 subunit of NF-κB, a substantial fraction of uninfected Vero cells displayed confined nuclear p65 staining, possibly indicating higher basal levels of NF-κB activation in this cell type. Nonetheless, infection of HSV-1 or HSV-2 resulted in substantially and significantly increased percentages of cells with nuclear p65. Surprisingly, we noticed that most of the p65 protein in HSV-1-infected Vero cells concentrates at the perinuclear area at 16 hpi, with only a minority effectively entering the nucleus, which contrasts the massive nuclear import of p65 in HSV-2-infected cells (Fig. 3D). Notably, the distinctive distribution of p65 was consistently seen at 16 hpi in Vero cells infected with other wild-type strains of HSV-1 and HSV-2, namely, HSV-1 KOS and HSV-2 MS (data not shown), indicating that these phenotypes are species-dependent but strain-independent. Still, EMSAs revealed that both HSV-1 and HSV-2 infection of Vero cells result in the generation of activated NF-κB that binds to κB-binding sites *in vitro* (Fig. 3E).

**FIG 4:**
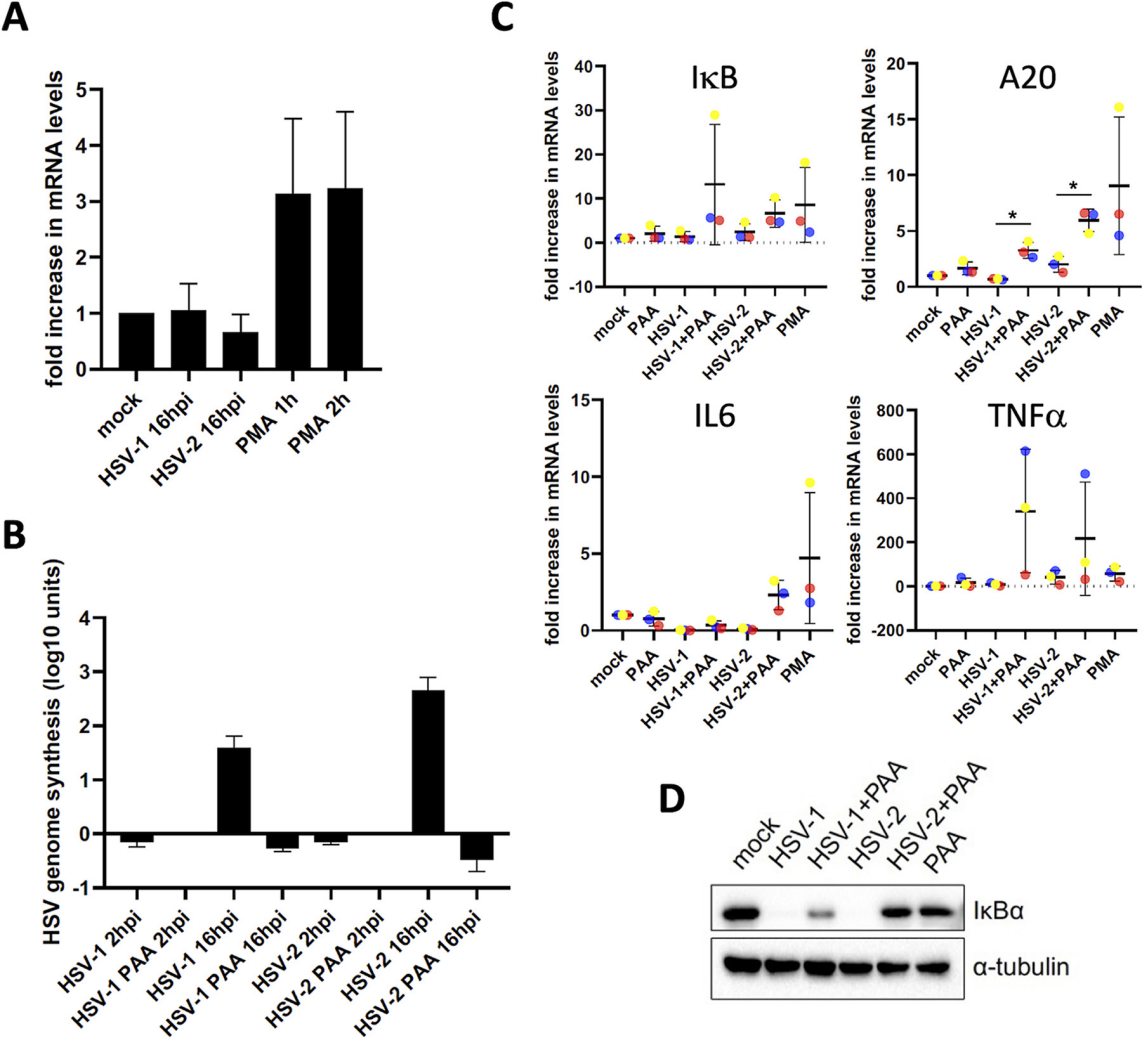
HSV-1- and HSV-2-induced NF-κB activation in Vero epithelial cells does not lead to NF-κB-dependent host gene expression. (A) RT-qPCR-based evaluation of IκBα mRNA levels in Vero cells at 16 hpi with HSV-1 or HSV-2 (MOI of 10 PFU/cell) or exposed to PMA for 1 or 2 h (20 ng/mL). The graph represents the mean and standard deviation of the relative fold change compared to the mock condition out of three independent repeats (transcript levels were normalized to 18S rRNA). (B) qPCR-based analysis of HSV-1 and HSV-2 genome replication in the presence or absence of the viral DNA polymerase inhibitor PAA (400 μg/mL) at 2 hpi and 16 hpi (MOI of 10 PFU/cell). Vero cells were pretreated with PAA for 30 min before virus inoculation, and the inhibitor was kept throughout the infection. The graph shows the mean and standard deviation (in log_10_ units, based on three independent repeats) of the relative fold change in viral DNA copies compared to the genome copies found in PAA-treated HSV-1- and HSV-2-infected Vero cells at 2 hpi (data were normalized to the host genome using the β-2-microglobulin (B2M) gene). Values were relative to PAA-treated PRV-infected cells at 2 hpi. (C) RT-qPCR-based quantitation of mRNA loads of the hallmark NF-κB-dependent IκBα, A20, TNF-α, and IL-6 genes in HSV-1- and HSV-2-infected Vero cells at 16 hpi (MOI of 10 PFU/cell), either or not treated with PAA (400 μg/mL, starting 30 min prior to inoculation and kept during infection). PMA treatment (1 h, 20 ng/mL) served as a positive control. The graphs show the means and standard deviations (three independent repeats) of the relative fold differences in mRNA levels versus the mock condition (transcript levels were normalized to 18S rRNA). Different colors differentiate the values obtained in each of the repeats of the experiment. Asterisks indicate statistically significant differences (*, *P* < 0.05) (D) Western blot analysis of the IκBα protein in HSV-1- and HSV-2-infected Vero cells at 16 hpi (MOI of 10 PFU/cell), treated or not with PAA (400 μg/mL). Vero cell monolayers were pretreated with PAA 30 min prior to infection, and PAA was maintained in the culture medium throughout the infection. All assays were independently repeated three times.

Notwithstanding and analogous to what we found for FHV-1 and PRV (Fig. 2E) (9), activation of NF-κB does not result in detectable expression of the hallmark NF-κB-dependent gene IκBα in infected Vero cells (Fig. 4A). This repressive effect of HSV-1 and HSV-2 infection on NF-κB-dependent gene expression in Vero cells was confirmed for three additional NF-κB-driven genes, namely, A20, tumor necrosis factor alpha (TNF-α), and interleukin-6 (IL-6), further highlighting that herpes simplex viruses inhibit NF-κB-dependent host gene expression in infected Vero cells (Fig. 4C). In FHV-1- and PRV-infected epithelial cells, inhibition of viral genome replication using PAA consistently restored NF-κB-dependent gene expression. In line with this, treatment with PAA, which effectively inhibits HSV-1/-2 genome replication (Fig. 4B), resulted in increased expression of NF-κB-driven host genes in HSV-1- and HSV-2-infected Vero cells, except for IL-6 in HSV-1-infected cells (Fig. 4C). Although the PAA-induced increase in NF-κB-driven host gene expression reached statistical significance only for A20, it was reproducible and observed in each of the three independent repeats for IκB, A20, and TNF-α. This suggests that, also in HSV-1- and HSV-2-infected Vero cells, suppression of NF-κB-dependent IκBα gene transcription (at least in part) depends on replication of the viral genome. In line with this, PAA treatment resulted in substantial replenishment of IκBα protein levels in HSV-infected Vero cells, particularly for HSV-1 (Fig. 4D). In summary, the interaction of the simplex viruses HSV-1 and HSV-2 with the NF-κB pathway in Vero epithelial cells is generally reminiscent of that observed for the varicella viruses PRV and FHV-1 in porcine and feline epithelial cells. Intriguingly, some aspects of this interaction appear to differ between HSV-1 and HSV-2, as exemplified by the distinctive subcellular location of the NF-κB p65 subunit in infected Vero cells.

## DISCUSSION

Interfering with the innate immune responses that are triggered by activation of the NF-κB pathway in infected epithelial cells represents a common capacity of different alphaherpesviruses, as demonstrated in the current and previous studies (9, 10, 12, 13). The preservation of this ability across different alphaherpesviruses emphasizes the plausible relevance of this suppression for alphaherpesviral infection fitness, at least in epithelial cells, which represent important target cells for these viruses. We speculate that suppression of NF-κB-dependent defense mechanisms, very likely in combination with the shutoff of other central antiviral signaling nodes, may contribute to a more stealth-like mode of virus spread, e.g., toward sensory neurons at infected epithelial barriers. This hypothesis is supported by the number of NF-κB-controlled genes that encode factors that may locally restrict virus fitness in epithelial cells (e.g., beta interferon [IFN-β]) or elicit more systemic antiviral responses (e.g., TNF-α, IL-6) (35–38).

Of note, in spite of the apparent ability and possible need of these viruses to prevent NF-κB-driven responses, it is intriguing why most of the alphaherpesviruses that we studied appear to mainly suppress the NF-κB cascade transcriptionally, and not upstream in the signaling axis. Recently, we reported that the PRV-induced shutoff of NF-κB-dependent genes concurred with the decrease of prespliced cellular housekeeping transcripts, with both processes relying on the onset of viral genome replication. This was associated with a nuclear relocation of essential host factors of the cellular transcription machinery, the RNA polymerase II and the TATA box-binding protein (TBP), to the viral replication compartments (VRCs), away from the host (marginalized) chromatin (13). In line with this, repression of cellular transcription and recruitment of host transcription factors to VRCs have also been documented during HSV-1 infection (39–41). Thus, if the persistent activation of the NF-κB cascade by these viruses in epithelial cells mainly takes place when the virus-induced broad host transcription shutoff is active, e.g., as described in PRV infection (13), it is reasonable to hypothesize that the inhibition of upstream steps in the NF-κB pathway may not constitute a major evolutionarily selective pressure during alphaherpesvirus evolution to further block NF-κB-driven responses. The fact that NF-κB transcription factors are able to interact *in vitro* with κB DNA sequences in epithelial cells infected with PRV, FHV-1, HSV-1, and HSV-2 (and to a much lesser extent BoHV-1) (current study; 13) further supports the idea that the lack of NF-κB-dependent gene expression is in most cases likely attributed to cellular gene transcription defects. However, deficient *in vivo* interaction of NF-κB dimers with κB sites at gene promoters cannot be ruled out, which for instance, could be explained by recruitment of NF-κB p65 within VRCs, as described for PRV (13), or the accumulation of p65 at perinuclear regions, as observed in HSV-1-infected Vero cells in the current study. Our results suggest that initiation of viral genome replication seems to be a shared determinant of the repression of NF-κB-dependent gene transcription in infected epithelial cells with PRV, FHV-1, and HSV-1/-2 (current study; 13). Future research will clarify exactly how viral DNA replication and/or viral replication-associated events negatively affect NF-κB transcription activity and, at least in the case of PRV (13), cellular transcription in general. Such insights may help to design therapeutic strategies for pathologies that are associated with exacerbated NF-κB activation, such as different types of cancer and inflammatory diseases (42, 43).

BoHV-1 looks like an exception within the *Alphaherpesvirinae*, at least in the cohort of viruses used in the current study, in terms of its interaction with the NF-κB pathway in epithelial cells. The apparent absence of prominent NF-κB activation in BoHV-1-infected epithelial cells may reflect that these cells fail to efficiently detect BoHV-1 via sensors that trigger the NF-κB pathway. Conversely, it is also possible that BoHV-1 actively suppresses such detection and/or early events in the NF-κB signaling either at or upstream of IκBα protein degradation. The fact that alphaherpesviruses that are evolutionarily more distinct—such as PRV, FHV-1, and HSV-1/-2—show a largely similar interaction with the NF-κB pathway, whereas BoHV-1 that is closely related to PRV behaves differently, suggests that in bovines, there may have been a particular need to interact differently with this very conserved key innate signaling pathway and suppress its activation very early in the signaling cascade. Interestingly, a recent report showed that NF-κB is activated during and contributes to BoHV-1 entry in MDBK cells (44). Some others indicate that BoHV-1 triggers NF-κB activation upon virus penetration (45, 46). Hence, although speculative, it is possible that rapid NF-κB activation during BoHV-1 infection of MDBK cells contributes to virus entry but has also resulted in additional viral mechanisms to suppress the pathway to interfere with its antiviral effects.

In summary, the present study shows that several mammalian alphaherpesviruses display a largely similar mode of interaction with the NF-κB pathway in infected epithelial cells, consisting of activation of the pathway but repression of the consequent NF-κB-dependent gene expression, with the notable exception of BoHV-1, which appears to also interfere with activation of the pathway. The notion that all studied alphaherpesviruses interfere with productive activation of the NF-κB pathway in infected epithelial cells indicates that this feature may be an important aspect of their ability to hide from and suppress the host immune system.

## MATERIALS AND METHODS

### Cell cultures and viruses.

Bovine kidney epithelial (MDBK) cells (ATCC NBL-1; Bos taurus, cow) were cultured in Dulbecco’s modified Eagle medium (DMEM) supplemented with 4.5g/L d-glucose (DMEM GlutaMAX), 10% inactivated fetal bovine serum (FBS), 1 mM sodium pyruvate, and 50 μg/mL gentamicin. Swine testicle (ST) epithelial cells (ATCC CRL-1746; Sus scrofa, pig) were cultured in modified Eagle’s medium (MEM) supplemented with 10% inactivated FBS, 100 U/mL penicillin, 0.1 mg/mL streptomycin, 50 μg/mL gentamicin, and 1 mM sodium pyruvate. Feline kidney epithelial (CRFK) cells (ATCC CCL-94; Felis catus, cat) were cultured in MEM supplemented with 10% inactivated FBS, 100 U/mL penicillin, 0.1 mg/mL streptomycin, and 1 mg/mL lactalbumin. Primate kidney epithelial Vero cells (ATCC CCL-81; Cercopithecus aethiops, African green monkey) were cultured in MEM supplemented with 10% FBS, 100 U/mL penicillin, 0.1 mg/mL streptomycin, and 50 μg/mL gentamicin.

BoHV-1 wild type (WT) Cooper strain was propagated and titrated on MDBK cell monolayers. PRV WT Kaplan strain was grown and titrated on ST cells. FHV-1 WT C27 strain was propagated and titrated on CRFK cells. Virus stocks of HSV-1 WT F strain and HSV-2 WT 333 strain were grown and titrated on Vero cell monolayers. All virus stocks were titrated by 10-fold serial dilution assays. Unless another multiplicity of infection (MOI) is specifically indicated, confluent epithelial cell monolayers were infected with 10 PFU per cell throughout the study.

### Inducers and inhibitors.

NF-κB inducer phorbol 12-myristate 13-acetate (PMA) was purchased from Enzo Life Sciences (catalog number BML-PE160-0005). Viral DNA polymerase inhibitor phosphonoacetic acid (PAA) was obtained from Sigma-Aldrich (catalog number 284270).

### Bright-field microscopy.

Live cells cultured in 6-well plates were visualized using a CKX41 inverted microscope (Olympus), and images were captured using INFINITY software, at the indicated time points.

### Western blotting.

Cell lysates were collected in ice-cold lysis buffer consisting of TNE (Tris-NaCl EDTA) buffer, pH 7.5, and 1% Nonidet P-40 (catalog number 37129000; Roche) in the presence of the protease inhibitor cocktail (cOmplete mini EDTA free; catalog number 11836170001; Roche). The procedure for SDS-PAGE and Western blotting is extensively described in Deruelle et al. (47). Blots were blocked in 5% (wt/vol) nonfat dry milk diluted in 0.1% phosphate-buffered saline (PBS)-Tween 20 (PBS-T) for 1 h at room temperature. The primary antibodies, the mouse monoclonal anti-IκBα antibody (clone L35A5; 1:1,000 dilution; catalog number 4814; Cell Signaling Technology), and the mouse monoclonal horseradish peroxidase (HRP)-conjugated anti-α-tubulin antibody (clone DM1A; 1:2,000 dilution; catalog number ab40742; Abcam) were incubated overnight at 4°C diluted in blocking buffer. After three 10-min washing steps with PBS-T, IκBα blots were incubated with HRP-conjugated goat anti-mouse IgG secondary antibody (1:2,000 dilution; catalog number P0447; Dako) for 1 h at room temperature. Protein bands were visualized via chemiluminescence, using either ECL Plus substrate (GE Healthcare) or SuperSignal West Femto maximum-sensitivity substrate (Thermo Scientific), with a ChemiDoc MP imaging device (Bio-Rad).

### Immunofluorescence assay.

Cell monolayers were rinsed once with 1 mL of phosphate-buffered saline (PBS) containing calcium and magnesium before fixation with 4% paraformaldehyde for 15 min at room temperature. After removal of the paraformaldehyde, cells were incubated with blocking/permeabilization solution (consisting of 5% inactivated FBS and 0.3% Triton X-100 diluted in PBS) for 1 h at 37°C. For NF-κB p65 detection, the mouse monoclonal anti-p65 antibody (clone L8F6; 1:400 dilution; catalog number 6956; Cell Signaling Technology) was diluted in incubation buffer, composed of 1% (wt/vol) bovine serum albumin (BSA) and 0.3% Triton X-100 diluted in PBS, and incubated overnight at 4°C. Mouse monoclonal antibodies for detection of BoHV-1 gC and gD (1:200 dilution) (kindly provided by G. Meyer [48]), for detection of and the mouse monoclonal anti-PRV gD antibody (clone 13D12; 1:50 dilution) (49), were diluted in incubation buffer and incubated for 1 h at 37°C. For the detection of FHV-1, anti-feline herpesvirus type 1/feline viral rhinotracheitis (FHV-1/FVR) polyclonal antiserum conjugated to fluorescein isothiocyanate (FITC) (Veterinary Medical Research and Development [VRMD], Pullman, WA, USA; catalog number CJ-F-FVR-10ML) was used. The slides were incubated with 300 μL of undiluted antiserum overnight at 4°C. For detection of gB of HSV-1/HSV-2 or FHV-1, a mouse monoclonal anti-HSV1/2 gB antibody (10B7 clone, 1:200 dilution; catalog number ab6506; Abcam, Cambridge, UK) or a mouse monoclonal anti-FHV-1 gB antibody (kindly provided by Yukinobu Tohya, University of Tokyo) was used, respectively, and antibodies were diluted in incubation buffer and incubated overnight at 4°C. The unbound primary antibody was removed through three washing steps with PBS, and afterwards, cells were incubated with fluorochrome-conjugated goat anti-mouse IgG secondary antibodies (1:200 dilution; Invitrogen) for 1 h at 37°C. Cell nuclei were counterstained using Hoechst 33342 (1:200 dilution; catalog number H3570; Invitrogen) for 10 min at room temperature. Images of NF-κB p65 immunofluorescence assays were taken using a Leica SPE confocal microscope, and images of those corresponding to viral glycoproteins were taken using a Thunder imaging system (Leica). In both cases, images were analyzed using ImageJ software (NIH, USA).

### NF-κB electrophoretic mobility shift assay.

Five million cells were lysed for 30 min in ice-cold electrophoretic mobility shift assay (EMSA) lysis buffer composed of 20 mM HEPES (pH 7.9), 350 mM NaCl, 1 mM MgCl_2_, 0.5 mM EDTA, 0.15 mM EGTA, 20% glycerol, 1% Nonidet P-40 (NP-40), 0.5 mM dithiothreitol (DTT), and protease inhibitor cocktail (1 tablet/10 mL of EMSA lysis buffer, cOmplete mini EDTA free; catalog number 11836170001; Roche) prior centrifugation at 14,000 × *g* for 10 min. Then, 1 mg of poly(dI-dC) (catalog number P4929; Sigma-Aldrich) and EMSA binding buffer (75 mM NaCl, 15 mM Tris HCl [pH 7.5], 1.5 mM EDTA, 1.5 mM DTT, 7.5% glycerol, 0.3% NP-40, 20 mg/mL BSA) were mixed with 10 mg of cell lysate in a total reaction volume of 9 μL for 20 min at 4°C. Next, 1 μl of [32P]-labeled double-stranded oligonucleotides (5′-TCAACAGAGGGGACTTTCCGAGAGGCC-3′) containing the underlined intronic κB site corresponding to the Igκ gene was added to the reaction mixture and incubated for 20 min at room temperature. Then, 4% native polyacrylamide gels were employed to separate the samples, which were later dried and exposed to film on a phosphor screen. NF-κB/κB complexes were visualized using Image Quant software.

### RNA isolation and reverse transcription.

Isolation of total RNA was carried out using the RNeasy minikit (catalog number 74104; Qiagen) following the manufacturer’s instructions, starting from approximately 2 million cells. Later, RNA yields were subjected to DNase I treatment (RNase free; catalog number M0303S; New England Biolabs) for 10 min at 37°C. The addition of EDTA (up to 5 mM) (catalog number AM9260G; Invitrogen) and the subsequent incubation at 75°C for 10 min were performed to inactivate DNase I activity. Next, 500 ng of DNase I-treated RNA was converted into cDNA through a reverse transcription (RT) reaction using the iScript cDNA synthesis kit (catalog number 1708891; Bio-Rad) in a final volume of 20 μL. The single-step reverse transcription consisted of 5 min at 25°C (priming), 20 min at 46°C (reverse transcription), and 1 min at 95°C (inactivation). Then, 1 μL of the resulting cDNA solution was subjected to analysis via real-time quantitative PCR (RT-qPCR).

### DNA isolation for viral genome quantitation.

Infected cell monolayers (ca, 2 × 10^6^ cells) were rinsed twice with PBS containing calcium and magnesium prior to treatment with sodium citrate buffer (pH 3.0; 40 mM sodium citrate, 10 mM KCl, and 135 mM NaCl) for 2 min at room temperature, to remove the nonentered virus particles. Later, cells were washed three additional times with complete PBS before sample collection. Cell pellets were immediately frozen at −20°C. The DNeasy blood and tissue kit (catalog number 69504; Qiagen) was used to isolate total DNA, following the manufacturer’s protocol. DNA yields were eluted in a final volume of 200 mL. A total of 1 μL of the DNA elution constituted the template for RT-qPCR analysis.

### Real-time quantitative PCR.

qPCR assays were conducted using SYBR green PCR master mix (catalog number 4309155; Thermo Fisher Scientific) using the primer DNA oligonucleotides (Integrated DNA Technologies) described in Tables 1 and 2, in a final volume of 20 mL. qPCRs were launched in MicroAmp Fast optical 96-well reaction plates (catalog number 4346906; Thermo Fisher Scientific) using a StepOnePlus real-time PCR system (catalog number 4376600; Thermo Fisher Scientific). Samples were evaluated in duplicates and analyzed by the double-delta threshold cycle method. Determination of viral genome replication was based on the detection of FHV-1, HSV-1, and HSV-2 US3 open reading frames, and as a normalization control, the host genomes were quantitated by targeting the beta-2-microglobulin (B2M) gene. For transcript-level quantification, mRNA loads corresponding to NF-κB-dependent genes IκBα, A20, TNF-α, and IL-6 were normalized with the levels of 18S rRNA, set as the housekeeping gene. When primer pair sequences were not obtained from previous studies, oligonucleotides were designed using PrimerBLAST software (NIH, USA) or the PrimerQuest tool (Integrated DNA Technologies).

**TABLE 1:** Primer DNA oligonucleotide sequences targeting viral US3 open reading frames (ORFs) used to determine intracellular viral DNA loads^*a*^

Target gene	GenBank ID	Forward sequence (5′–3′)	Reverse sequence (5′–3′)
FHV-1 US3 ORF; 117698–120188)	NC_013590.2	CTC CCG CAA CCA TCT TCG TA	TTT AGA AGT CGG GTC GCC TG
HSV-1 US3 ORF	NC_001806.2	CCT AAG CGC CGT TGA CTA CA	AAA GTC CCC CAG GCA AAT GT
HSV-2 US3 ORF	NC_001798.2	CGA GAA CAT CTG TCT GGG GG	CGA TCC CGT AAT GGA AGG GG
B2M gene (Felis catus)	NC_018728.3	TCA CAC CCG AAG GTG ACA AG	CAG ACA GTC GAG GGG GAC TA
B2M gene (*Chlorocebus* sp.; 102721416–102725526)	NW_023666048.1	TGG GTT GAT CCG CTT AGG AAC	CTG CCT CGA TCT ACA CCC AC

a ORFs, open reading frames.

**TABLE 2:** Primer DNA oligonucleotide sequences used to study NF-κB-dependent gene expression in CRFK and Vero cell lines

Target gene	GenBank ID	Forward sequence (5′–3′)	Reverse sequence (5′–3′)
IκBα mRNA (Felis catus)	XM_003987542.5	CTG CAG AAT TCC GAC CTC GT	GCT GCT GCT GTA TCC GTG TA
18S rRNA (Felis catus) (50)		GAC GAC CCA TTC GAA CGT CT	TGC TGC CTT CCT TGG ATG TG
IκBα mRNA (*Chlorocebus* sp.)	XM_007986484.2	ACC TCT GTG GGG TTT TTG GAC	TGA AAG GTC TAA CAC TCC TGG C
TNFα mRNA (*Chlorocebus* sp.)	XM_038005847.1	ACC AGC TAA GAG GGA GAG AA	GTC AGT ATG TGA GAG GGA GAGA
A20 mRNA (*Chlorocebus* sp.)	XM_008006880.2	GCC CAG GAA TGC TAC AGA TAC	AGT GGA ACA GCT CGG ATT TC
IL6 mRNA (*Chlorocebus* sp.)	XM_007981902.2	CTC CCA GGA GAA GAT TCC AAA G	CGT CGA GGA TGT ACC GAA TTT
18S rRNA (*Chlorocebus* sp.) (51)		GTA ACC CGT TGA ACC CCA TT	CCA TCC AAT CGG TAG TAG CG
